# Impact of Coronavirus Disease 2019 Pandemic on Crowding: A Call to Action for Effective Solutions to “Access Block”

**DOI:** 10.5811/westjem.2021.2.49611

**Published:** 2021-07-16

**Authors:** Gabriele Savioli, Iride Francesca Ceresa, Roberta Guarnone, Alba Muzzi, Viola Novelli, Giovanni Ricevuti, Giorgio Antonio Iotti, Maria Antonietta Bressan, Enrico Oddone

**Affiliations:** * Fondazione IRCCS Policlinico San Matteo, Department of Emergency Medicine, Pavia, Italy; † Fondazione IRCCS Policlinico San Matteo, Medical Direction, Pavia, Italy; ‡ University of Pavia, Department of Drug Science, Pavia, Italy; § Saint Camillus International University of Health Sciences, Department of Drug Science, Rome, Italy; ¶ Fondazione IRCCS Policlinico San Matteo, Intensive Care Unit, Pavia, Italy; || University of Pavia, Department of Public Health, Experimental and Forensic Medicine, Pavia, Italy; # University of Pavia, Department of Clinical-Surgical, Diagnostic and Pediatric Sciences, Pavia, Italy

## Abstract

**Introduction:**

Healthcare patterns change during disease outbreaks and pandemics. Identification of modified patterns is important for future preparedness and response. Emergency department (ED) crowding can occur because of the volume of patients waiting to be seen, which results in delays in patient assessment or treatment and impediments to leaving the ED once treatment is complete. Therefore, ED crowding has become a growing problem worldwide and represents a serious barrier to healthcare operations.

**Methods:**

This observational study was based on a retrospective review of the epidemiologic and clinical records of patients who presented to the Foundation IRCCS Policlinic San Matteo in Pavia, Italy, during the coronavirus disease 2019 (COVID-19) outbreak (February 21–May 1, 2020, pandemic group). The methods involved an estimation of the changes in epidemiologic and clinical data from the annual baseline data after the start of the COVID-19 pandemic.

**Results:**

We identified reduced ED visits (180 per day in the control period vs 96 per day in the pandemic period; P < 0.001) during the COVID-19 pandemic, irrespective of age and gender, especially for low-acuity conditions. However, patients who did present to the ED were more likely to be hemodynamically unstable, exhibit abnormal vital signs, and more frequently required high-intensity care and hospitalization. During the pandemic, ED crowding dramatically increased primarily because of an increased number of visits by patients with high-acuity conditions, changes in patient management that prolonged length of stay, and increased rates of boarding, which led to the inability of patients to gain access to appropriate hospital beds within a reasonable amount of time. During the pandemic, all crowding output indices increased, especially the rates of boarding (36% vs 57%; P < 0.001), “access block” (24% vs 47%; P < 0.001), mean boarding time (640 vs 1,150 minutes [min]; P 0.001), mean “access block” time (718 vs 1,223 min; P < 0.001), and “access block” total time (650,379 vs 1,359,172 min; P < 0.001).

**Conclusion:**

Crowding in the ED during the COVID-19 pandemic was due to the inability to access hospital beds. Therefore, solutions to this lack of access are required to prevent a recurrence of crowding due to a new viral wave or epidemic.

## INTRODUCTION

Coronavirus disease 2019 (COVID-19) is an acute respiratory infectious disease caused by the novel coronavirus severe acute respiratory syndrome coronavirus 2 (SARS-CoV-2). SARS-CoV-2 is dissimilar to other coronaviruses that usually spread in humans: it is particularly pathogenic in humans and is associated with high mortality rates.^1^ Viruses that cause respiratory tract infections can exacerbate chronic lung disease, requiring visits to the emergency department (ED) and hospitalization.^2^–^8^ Therefore, identifying viruses and monitoring the severity of their effects will remain major scientific and clinical endeavors.

Healthcare utilization changes during infectious disease outbreaks. Identifying the patterns of change is important for future preparedness and response. The effects of infectious disease epidemics on healthcare utilization depend on the characteristics of the infection.^9^–^13^ Thus, epidemics have major effects on the healthcare system, including crowding. Crowding in the ED can occur because of the volume of patients waiting to be seen (input), delays in patient assessment or treatment (throughput), or impediments to leaving the ED once treatment has been completed (output).^14^ Emergency department crowding has become a growing problem globally that represents a serious impediment to healthcare utilization. Crowding is the product of several internal and external factors, including insufficient access to hospital beds and shortages of hospital staff. Studies reported that crowding can result in a higher number of adverse events, increased morbidity and mortality, prolonged length of stay (LOS), and reduced healthcare quality. 15

Currently, the most frequent cause of ED crowding is access block. The Australasian College for Emergency Medicine (ACEM) defines access block as “the situation where patients are unable to gain access to appropriate hospital beds within a reasonable amount of time, no greater than 8 hours”; it further defines crowding as “the situation where ED function is impeded by the number of patients waiting to be seen, undergoing assessment and treatment, or waiting for departure, exceeding the physical or staffing capacity of the department.”^16^ The effects of the COVID-19 pandemic on the availability of emergency services and ED crowding have not been evaluated. We conducted a large, retrospective observational study to compare the demographic and clinical data of patients after the start of the pandemic with data for patients who visited the ED in the corresponding period in the prior two years, as well as the period preceding the outbreak. We found that crowding increased as measured using throughput and output indices. The specific hypotheses were as follows:

1. the number of patients who presented to the ED decreased after the COVID-19 outbreak regardless of age and gender;
2. the modes of ED access (eg, ambulance, spontaneous), the codes for priority for medical examination, and the exit codes^17^–^21^ (severity codes for discharge determined through clinical criteria assigned to patients by the attending emergency physicians who maintained the same classification as in triage) for severity changes after the outbreak reflect more serious illness and patients requiring high-intensity care;
3. the marked reduction in some access types (such as access for minor trauma and minor signs and symptoms) was accompanied by a homogeneous reduction in other access types;
4. throughput (such as ED LOS) and output crowding indices (such as rate of access block, total access block time, and percentage of patients who left without being seen) have been made worse by the COVID-19 outbreak;
5. clinical outcomes, such as admission and mortality rates, were worsened by the outbreak; and
6. visits attributable to the COVID-19 outbreak accounted for the majority of ED visits. The final objectives of this study were to estimate the rate of ED visits attributable to the outbreak and guide the planning of strategies for managing ED access after the outbreak of transmittable respiratory diseases.

**Population Health Research Capsule**

What do we already know about this issue?

*Epidemics change the way patients use health services, leading to crowding that in turn leads to worse outcomes including increased adverse events and mortality*.

What was the research question?

*How did the COVID 19 pandemic change the use of healthcare and emergency departments (ED) and what were the consequences?*

What was the major finding of the study?

*We found a decrease in ED access, while crowding increased due to throughput and output factors, mainly due to exit block such as prolonged boarding*.

How does this improve population health?

*The problem of crowding, and in particular the exit block, must be solved at its root to improve patient care*.

## METHODS

### Study Design

This observational study was based on a retrospective review of the epidemiologic and clinical records of patients visiting Foundation IRCCS Policlinic San Matteo in Pavia, Italy, during the COVID-19 outbreak (February 21–May 1, 2020, pandemic group). We set as control periods, in which data on ED accesses were collected, the entire January–May periods in 2018 and 2019 (years before the pandemic) and the time span between January 1–February 20, 2020, because no emergency was declared before February 21, 2020. We extracted data using PiEsse software (PiEsse SRL, Latina, Italy). The methods included estimating the changes in epidemiologic and clinical data from the annual baseline data after the start of the COVID-19 pandemic. At the time of ED admission, patients provided informed consent for the processing of their data for medical and research purposes.

### Endpoints

We aimed to assess the changes in the use of emergency resources after the COVID-19 outbreak in terms of ED visits. The key secondary aim was to define the characteristics of the population that visited our ED during the pandemic, including gender, age, and method of ED access. Other examined outcomes included the causes of ED visits during the pandemic; crowding indices such as ED LOS, total access block time, and rate of access block; clinical outcomes such as admission and mortality rates; and the proportion of ED visits attributable to COVID-19.

### Inclusion and Exclusion Criteria

All non-pediatric patients (>14 years old) who visited the ED during the study periods were eligible for inclusion. Children under the age of 14 were not included as our ED is for adults. We treat children only if the reason for access is trauma; children who present for other medical reasons are referred to another ED. The same admission criteria apply to gynecological and ophthalmic emergencies: these patients are referred to specialized EDs separate from ours.

### Study Population

For each patient, we collected demographic data (gender and age); vital parameters (blood, heart rate, oxygen saturation, Glasgow Coma Scale, respiratory rate); signs and symptoms; waiting time; LOS in the ED; mode of presentation to the ED; priority codes for medical examination; exit codes for severity; total access block time; and rate of access block. All medical records were accurately viewed and evaluated, and all computed tomography data were thoroughly reviewed. In this study, the pandemic group consisted of 6728 consecutive patients who presented to the ED between February 20–May 1, 2020. The time periods span from January 1–May 1, 2018, and January 1–May 1, 2019. We used January 1–February 20, 2020, as reference intervals.

### Measurement of Crowding

Several indices to measure crowding have been proposed.^11^ The most commonly used indices can be grouped as follows:

- Input crowding indices: waiting times, number of patients visiting the ED, and disease severity and complexity (eg, number of patients at each acuity level), and the number of patients who left without being attended to;
- Throughput crowding indices: LOS;
- Output crowding indices: mean number or percentage of admissions, patients in the ED (number or percentage), access block and boarding (mean number or percentage of patients who experienced these), and access block or boarding times (such as the total access block time).

“Waiting time” is defined as the total time from initial registration/triage to first being seen by a doctor. The overall LOS in the ED is the time from arrival at triage or registration until discharge or transfer to a ward. This variable reflects the total patient experience, including care and waiting. Access block is defined as a greater than eight hours duration in the ED from presentation to admission.^22^–^23^ Total access block time thus represents the aggregate duration of access block for all patients studied.^24^ Boarding is defined as a greater than six hours duration in the ED from medical examination to admission.^25^ Thus, the total boarding time represents the aggregate duration of boarding for all patients studied.^22^–^25^

### Statistical Analysis

We performed statistical analyses using the appropriate logistic multivariate regression models to test the association between the risk of overtime for selected time variables, to account for crowding, and the pandemic period. Continuous variables were expressed as the mean and the standard error of the mean, whereas qualitative variables were expressed as the number of observations and appropriate proportions. We made comparisons between two groups of continuous variables using Student’s t-tests, whereas associations between qualitative variables were compared using the χ^2^ test. Moreover, the test of proportions was used to assess the differences in ED mortality between periods. All tests were two-tailed, and the significance level was set at an alpha of 0.05 (statistical significance at *P* < 0.05). The analyses were performed using STATA software: release 14 (StataCorp, LLC, College Station, TX).

## RESULTS

### Use of Emergency Resources

Total and daily access between February 20–May 1, 2020 (96 patients per day) was approximately 50% lower than the control period (180 attenders per day); ED visits related to seasonal flu increased (five per day in control period vs 17 per day in the pandemic period; *P* < 0.001). Regardless of gender, the number of ED visits was lower during the pandemic period than during the other periods (6,729 vs 8,714–12,543). During the pandemic, a slight but statistically significant male predominance was observed among patients who visited the ED (3,660 vs 3,069, *P* < 0.001. We divided the population into age groups as follows: <20; 20–29; 30–39; 40–49; 50–59; 60–69; 70–79; and ≥80 years. During the pandemic we observed reductions in the number of ED visits among all age groups, particularly among patients younger than 30 (*P* < 0.001; Table 1).

### Characteristics of Patients Who Visited Our ED During the Pandemic

The mode of arrival to the ED markedly changed during the pandemic. Whereas 60–70% of patients typically arrived to the ED using their own transportation prior to the pandemic, only 40% of patients arrived via autonomous means during the pandemic (*P* < 0.001). During the pandemic, a greater need for medical care and higher intensity of care were observed. Conversely, fewer patients required low-intensity care (31.2% vs 25.2%; *P* < 0.001). During the pandemic, the vital signs of the patients had deteriorated. Compared with the control groups, patients visiting during the pandemic displayed reduced oxygen saturation, higher rates of tachycardia, and lower systolic blood pressure values (see Table 2). We then compared the periods according to the percentage of patients with initial hemodynamic impairment and defined these patients as the those with impaired oxygen saturation (<95%), tachycardia (heart rate > 110 beats per minute), or arterial hypotension (systolic blood pressure < 90 millimeters mercurymmHg); we found that during the pandemic, patients were more likely to present with an initial hemodynamic impairment.

### Various Causes of ED Visits

During the pandemic, fewer patients visited the ED for minor medical issues (eg, dermatological conditions, otolaryngological diseases) and minor trauma (respectively: 29 access per day vs 10; 50 access per day vs 11; *P* <0.001 (Table 3). Visits because of work accidents also declined regardless of gender or age (7 vs 2 access per day; *P* <0.001), as did the proportion of patients with major trauma (1 vs 0 access per day; *P* <0.001), which was dramatically reduced. Access for other causes had an homogeneous reduction: this applies, for example, to patients with access for neurological symptoms (13 vs 7 access per day, *P* <0.001), and for chest pain (13 vs 7 access per day). Conversely the percentage of patients who reported fever symptoms at home was much higher (7 vs 16 access per day; *P* <0.001), whereas the proportion of patients who had fever at triage was unchanged.

### Crowding Indices

#### Input Indices

During the pandemic, a reduction in waiting time (from arrival at the ED until seen by a doctor) was observed for triage codes 5 (the lowest acuity code), 4, and 3, whereas for code 2, this reduction was not statistically significant, and for code 1 (the highest acuity code), only a small significant increase in waiting time was observed (66 vs 83 min; *P* < 0.001 (Tables 4 and S1).

#### Throughput Indices

During the pandemic the time spent in the ED increased, especially LOS (625 vs 314 min; *P* < 0.001. The prolongation of LOS in the pandemic period compared with that in the control periods remained statistically significant after adjustment for age, gender, priority code, and the need for moderate-to-high–intensity care (625 vs 314 min, P < 0.001 (Table 5).

#### Output Indices

During the pandemic, all crowding output indices increased, especially the rates of boarding (36% vs 57%; *P* < 0.001), access block (24% vs 47%; *P* < 0.001), mean boarding time (640 vs 1150 min; *P* <0.001), mean access block time (718 vs. 1223 min; *P* < 0.001), and access block total time (650,379 vs. 1,359,172 min; *P* < 0.001. The increased frequencies of boarding (percentage and total time) and access block (percentage and total time) in the pandemic period compared with that in the control periods remained statistically significant after adjustment for age, gender, priority code, and the need for moderate-to-high–intensity care (*P* < 0.001).

### Clinical Outcomes

During the pandemic, patients had worse exit codes (severity codes for discharge through clinical criteria assigned by the attending emergency physicians who maintained the same classification as in triage) and hospitalization rates (*P* < 0.001). The need for hospitalization increased from approximately 16% to 34% (*P* < 0.001). Importantly, although the total number of ED visits decreased, the number of deaths increased. In fact, we observed 115 deaths between February 21–May 1, 2020 (pandemic), while the number of deaths during the control period was 75. Considering the difference in patient numbers (6,729 during the pandemic period and 51,439 in the control period), we found mortality rates in the ED of 1.71 per 100 patients during the pandemic and 0.15 per 100 patients (*P* < 0.001) in the previous corresponding periods.

### Proportion of Visits Attributed to COVID-19

To assess the proportion of ED visits attributable to the pandemic, we analyzed patients with signs or symptoms that required a differential diagnosis for SARS-CoV-2 infection. The percentage of patients who visited the ED for relevant symptoms (fever or respiratory problems) was 30.47% during the pandemic period vs 9.62% during the control period (*P* < 0.001).

## DISCUSSION

### Use of Emergency Resources and Characteristics of Patients Who Visited Our ED During the Pandemic

The high number of deaths associated with the COVID-19 pandemic spurred civil authorities to implement measures to contain the virus. “Red zones” were created, including restrictions on citizens’ movements, business closures, and advisements to work from home when possible. Newscasts that constantly updated the spread and mortality of COVID-19 likely resulted in increased apprehension among the population.

As observed in previous studies examining changes in healthcare utilization according to disease severity,^26^–^27^ the results of this situation showed that the reduction in emergency care utilization was most prominent for low-acuity conditions (non-urgent; minor emergency; emergency requiring low-intensity care).^26^ The reduction in visits for high-acuity conditions (emergency requiring moderate-to-high–intensity care) was relatively small, despite the possibility of more serious consequences (late or missed diagnoses of some conditions, even serious ones, and time-dependent conditions such as heart attacks and strokes). The increased use of the ED by sicker patients was also evidenced by the higher prevalence of hemodynamically compromised patients. Examining the scale of ED visits for low-acuity conditions with little benefit from service use is important for both ensuring appropriate emergency surge capacity and providing evidence to redesign emergency services to decrease healthcare-related infections after disease outbreak.

### Various Causes of ED Visits

During the pandemic there was a net reduction in some reasons for ED visits such as minor trauma or minor medical issues, confirming the reduction of low-acuity visits. Although the percentage of patients who had febrile symptoms at home was much higher during the pandemic, the proportion of patients who had fever at triage was not increased. This is also likely attributable to the fact that body temperature has been measured in a greater number of patients during the pandemic (53.2% vs 12.7% before the pandemic).

Patients decide to use medical care after considering the risks and benefits. When patients have concerns about nosocomial infections, those with low-acuity diseases are less likely to visit the ED.^26^–^27^ Visits by patients with low-acuity conditions most strongly decrease when the risk of infection overwhelms the benefits of emergency service use. The rate of visits for serious conditions did not decline in the same manner. Even the inputs for high-acuity diseases, albeit stable in percentage terms, were reduced, although to a smaller degree. This is the case, for example, with presentations for chest pain and neurological disorders. This has been highlighted by some studies which reported an increase in late diagnoses.^47^–^50^ When fears of an epidemic spread and ED visits decrease, preparations for serious conditions must be focused, and patients with severe diseases should not face barriers to emergency care. This situation also underlines the need to consider “clean” or low-risk infectious pathways for the most serious reasons for ED visits.

### Crowding Indices

#### Causes of Crowding

Crowding of EDs has been reported for several decades. Our study found that input factors played a modest/ ambivalent role in crowding in this pandemic. ED crowding had two main causes: the worsening of output and throughput factors. With regard to output factors, crowding was caused by the access block phenomenon and in particular by an unprecedented need for care in medium- and high-intensity care units.^1^ In a study conducted prior to this pandemic, through tabletop simulations of a potential maxi-emergency, our research group had anticipated that such a scenario was possible. In particular, we had shown how wards with high- and medium-intensity care could most easily determine boarding time and access block. 17

We believe this increment of access block is attributable to the discrepancy between the immediate and sudden need for intensive care (ICU) beds and the number of ICU beds available on the basis of national and local historical needs. However, it is important to emphasize that all patients, even those in need of low-intensity care, have struggled against access block. Therefore, the lack of beds seems to be the main cause of access block. Our opinion is that EDs are crowded when hospitals are crowded. The waiting time for hospitalization was also prolonged because it was necessary to screen all patients before assigning them to a “clean” vs COVID-unit bed to ensure that infected (and perhaps asymptomatic) patients were not admitted to “clean” wards or wards in which the risk of infection had to remain low.

With regard to throughput factors, crowding has resulted from changes in the role of emergency physicians and EDs. Emergency departments are no longer merely where patients are sorted into specialist departments; patients are now treated and stabilized, and differential diagnostic tests are performed in the ED. This change in the level of care has been exacerbated in the pandemic because of the high number of critically ill patients who require stabilization before transfer to the hospital wards, and the change in patient management caused by the pandemic. In particular, the need for frequent checks, ventilatory therapies, nasal swabs and wait time for the result, the time taken for dressing and undressing by the medical and nursing staff, and the high burden of caring for patients who need ventilatory therapy mean that patients often cannot be autonomous, and because of the disease, relatives and other caregivers cannot stay to help them. As a result, the care burden on health workers has also increased.

In our opinion, the necessary doubling of patient flows (COVID-free flow and COVID flow) has also contributed to increases in work and crowding, which have doubled the work of the ED staff with the same amount of resources. In fact, nasal swabs (for serological tests), bedside chest radiographs, and bedside lung ultrasounds were obtained from all patients who awaited the results in a specific location separate from other inpatients in the ward. These necessary safety measures prolonged the processing time and LOS, together with frequent sanitation and the use of personal protective equipment by healthcare professionals. Thus, increased rates of boarding and access block during the pandemic affected all patients, including those who did not have COVID-19, despite the strong effort during the emergency peak to add approximately 300 beds for COVID-19 patients, 65 of which were dedicated to the ICU.

During the pandemic, the treatment of COVID-19 has progressively changed, particularly the indications for intubation. Early on, patients were intubated early; now alternative modes of support (eg, high-flow nasal cannula, non-invasive positive-pressure ventilation, awake proning) are recommended before intubation. Nevertheless, the need for medium- or high-intensity care persists, and COVID-19 wards are the departments that probably prolong boarding.

#### Possible Crowding Responses

Many researchers and societies have developed measures to prevent ED crowding and provide proper care for patients receiving emergency care. Interventions are categorized into input, throughput, and output controls. 29–33 However, measures to alleviate crowding and reduce access block are needed to prepare adequate responses for future pandemics.

Emergency preparedness for outbreaks of transmittable respiratory illness has scarcely focused on preventing crowding and protecting staff and patients. Rather, the focus has been on preparing emergency quarantine areas and isolating admission rooms. Crowding provides favorable conditions for transmission among patients in the ED through respiratory droplets, and prior research has recommended infection control measures such as case management, isolation, and planning for complex emergencies.^34^–^46^

To improve the practice of boarding patients, the American College of Emergency Physicians (ACEP) established a task force to develop a list of low-cost, high-impact solutions.^42^–^43^ One of the key solutions proposed by ACEP is the use of a full-capacity protocol. 41 Although this was an effective response, the need for effective solutions for reducing access block must be reiterated. Given the emergence of pandemics and other emergencies, we must emphasize that “access Block and ED overcrowding have created a dynamic tension and the future of emergency medicine will be determined by the resolution of this conflict.”^46^

### Clinical Outcomes, Like Admission and Mortality Rates, Were Made Worse by the Outbreak

The rates of more serious exit codes and the need for hospitalization were approximately twofold higher than those in the control periods. This illustrates the major impact of this pandemic on the healthcare system^1^–^7^ and simultaneously highlights the high rates of access block and boarding that occurred. A greater need for hospitalization, in this case nearly twofold higher than the historical requirement, resulted in a more rapid saturation of hospital beds. In addition, patients with greater disease severity require longer hospital stays.

### Visits Attributable to the COVID-19 Outbreak Accounted for the Majority of ED Visits

To assess the rate of ED visits attributable to the COVID-19 outbreak, we analyzed ED visits associated with symptoms compatible with SARS-CoV-2 infection because the clinical suspicion and symptoms cited by the patient determines access to the ED as opposed to the final diagnosis. Specifically, patients with respiratory symptoms and fever are sent to the ED for suspected COVID-19. Excluding such a diagnosis does not reduce the use of EDs.

This study confirmed that a higher number of patients visited the ED with febrile or respiratory symptoms during the pandemic, comprising approximately one-third of all ED visits. Of course, only a portion of these patients received a diagnosis of COVID-19 or required hospitalization. This indicates that following an outbreak, more patients with symptoms of milder respiratory illness use emergency resources, and more patients seek emergency care at an early stage. These findings should be considered when creating effective responses to epidemics or pandemics involving respiratory symptoms.

## CONCLUSION

This study identified a reduction in ED visits during the COVID-19 pandemic irrespective of age and gender, especially for low-acuity conditions. However, patients who visited the ED more frequently were hemodynamically unstable, more commonly exhibited abnormal vital signs, and more frequently required high-intensity care and hospitalization. During the pandemic, ED crowding dramatically increased, primarily because of increased visits by patients with high-acuity conditions, changes in patient management that prolonged lengths of stay, and increased rates of boarding and access block.

## Supplementary Information



## Figures and Tables

**Table 1:** Principal personal and emergency department presentation features of patients included in the study, by period of observation.

		Period*				

		Control	Pandemic	Difference	P^a^	Total
Total patients		51,439	6,729			58,168
Daily visits		180	96			276
Gender						
Male	n (%)	26,395 (51.34)	3,660 (54.39)	−22,735		30,055 (51.70)
Female	n (%)	25,014 (48.66)	3,069 (45.61)	−21,945	<0.001	28,083 (48.30)
Age group						
<20	n (%)	5,878 (11.43)	310 (4.61)	−5,568		6,188 (10.64)
20–29	n (%)	5,561 (10.82)	507 (7.53)	−5,054		6,068 (10.44)
30–39	n (%)	5,181 (10.08)	636 (9.45)	−4,545		5,817(10.01)
40–49	n (%)	6,676 (12.99)	874 (12.99)	−5,802		7,550 (12.99)
50–59	n (%)	6,754 (13.14)	1,019 (15.14)	−5,735		7,773 (13.37)
60–69	n (%)	5,703 (11.09)	936 (13.91)	−4,767		6,639 (11.42)
70–79	n (%)	6,946 (13.51)	1,100 (16.35)	−5,846		8,046 (13.84)
80+	n (%)	8,710 (16.94)	1,347 (20.02)	−7,363	<0.001	10,057 (17.30)
Transport						
Personal	n(%)	33,870 (65.88)	2,859 (42.49)	−31,011		36,729 (63.18)
Ambulance with volunteer personnel (paramedic)	n(%)	7,757 (15.09)	1,719 (25.55)	−6,038		9,476 (16.30)
Ambulance with specialized nurse	n (%)	8,483 (16.50)	1,986 (29.51)	−6,497		10,469 (18.01)
Ambulance with doctor	n (%)	1,022 (1.99)	143 (2.13)	−879		1,165 (2.00)
Other	n(%)	277 (0.54)	22 (0.33)	−255	<0.001	299 (0.51)
Triage priority						
5 code	n (%)	3,631 (7.05)	294 (4.36)	−3,337		3,924 (6.74)
4 code	n (%)	31,712 (61.71)	3,947 (58.68)	−27,765		35,659 (61.36)
3 code	n (%)	3,119 (6.06)	393 (5.83)	−2,726		3,511 (6.03)
2 code	n (%)	12,137 (23.61)	1,933 (28.73)	−10,204		14,068 (24.20)
1 code	n (%)	814 (1.57)	163 (2.41)	−651	<0.001	976 (1.67)
Outcome						
Discharge	n (%)	41,580 (80.88)	4,249 (63.14)	−37,331		45,829 (78.83)
Hospitalization	n (%)	8,393 (16.33)	2,277 (33.84)	−6,116		10,670 (18.35)
Transfer	n (%)	839 (1.63)	133 (1.98)	−706		972 (1.67)
Other	n (%)	597 (1.16)	70 (1.04)	−527	<0.001	667 (1.15)

* The considered pandemic period was February 21–May 1, 2020. The control period was the sum of the timespans January 1–May 1, 2018; January 1–May 1, 2019; and January 1–February 20, 2020.

a χ^2^ test.

**Table 2:** Principal heart function parameters at presentation for patients included in the study, by period of observation.

	Period*			

	Control	Pandemic	P	Total
Heart rate				
Observations	32,228	5,278		37,506
Mean (bpm)	83.94	86.26		84.26
SE	0.10	0.25	<0.001^a^	0.09
Heart rate >110 bpm				
No (%)	30,219 (93.8)	4,854 (92)	<0.001^b^	35,073 (93.5)
Yes (%)	2,009 (6.2)	424 (8.0)		2,433 (6.5)
O_2_ saturation				
Observations	32,113	5,273		37,386
Mean (%)	97.2	96		97. 0
SE	0.02	0.06	<0.001^a^	0.02
O_2_ saturation <95%				
No (%)	28,022 (87.3)	4.103 (77.8)		32,125 (85.9)
Yes (%)	4,091 (12.7)	1,170 (22.2)	<0.001^b^	5,261 (14.17)
Systolic blood pressure				
Observations	32,497	5,312		37,809
Mean (mm Hg)	138.5	137.5		138.4
SE	0.13	0.32	0.004^a^	0.12
Systolic blood pressure < 90 mm Hg				
No (%)	32,168 (98.99)	5,242 (98.68)		37,410 (98.94)
Yes (%)	329 (1.01)	70 (1.32)	0.043^b^	399 (1.06)

* The considered pandemic period was February 21 to May 1, 2020. The control period was the sum of the timespans January 1 to May 1, 2018; January 1 to May 1, 2019; and January 1 to February 20, 2020.

a t-test.

b χ^2^ test.

*bpm*, beats per minute; *SE*, standard error; *O_2_*, oxygen; *mm Hg*, millimeters of mercury.

**Table 3:** Selected reasons for access to emergency department for patients included in the study, by period of observation.

	Period*			

	Control	Pandemic	P^a^	Total
Minor medical issues				
No (%)	44,629 (86.8)	6,057 (90.0)		50,686 (87.2)
Yes (%)	6,780 (13.2)	672 (10)	<0.001	7,452 (12.8)
Minor trauma				
No (%)	39,692 (77.2)	5,954 (88.5)		45,646 (78.5)
Yes (%)	11,717 (22.8)	775 (11.5)	<0.001	12,492 (21.5)
Major trauma				
No (%)	51,182 (99.6)	6,725 (99.9)		57,907 (99.6)
Yes (%)	227 (0.4)	4 (0.1)	<0.001	231 (0.4)
Occupational accident				
No (%)	49,710 (96.7)	6,569 (97.6)		56,279 (96.8)
Yes (%)	1,699 (3.3)	160 (2.4)	<0.001	1,859 (3.2)
Disease with fever				
No (%)	49,790 (96.8)	5,572 (82.8)		55,362 (95.2)
Yes (%)	1,619 (3.1)	1,157 (17.2)	<0.001	2,776 (4.8)
Respiratory symptoms				
No (%)	48,085 (93.5)	5,836 (86.8)		53,921 (92.8)
Yes (%)	3,324 (6.5)	893 (13.3)	<0.001	4,217 (7.3)
Thoracic pain				
No (%)	47,227 (91.9)	6,136 (91.2)		53,363 (91.8)
Yes (%)	4,182 (8.1)	593 (8.8)	0.057	4,775 (8.2)
Neurologic disease				
No (%)	48,364 (94.1)	6,222 (92.5)		54,586 (93.9)
Yes (%)	3,045 (5.9)	507 (7.5)	<0.001	3,552 (6.1)

* The considered pandemic period was February 21–May 1, 2020. The control period was the sum of the timespans January 1–May 1, 2018; January 1–May 1, 2019; and January 1–February 20, 2020.

a χ^2^ test.

**Table 4:** Selected time variables accounting for crowding, by period.

	Period*	Observations	Mean	Standard error	**Sum**	P^a^
Wait time (min)						
	Control period	51,405	83	0.36	**-**	
	Pandemic	6,729	66	0.98	**-**	<0.001
LOS (min)						
	Control period	51,405	314	1.84	**-**	
	Pandemic	6,729	625	11.36	**-**	<0.001
Process time (min)						
	Control period	51,405	231	1.81	**-**	
	Pandemic	6,729	560	11.30	**-**	<0.001
Access block time per patient^b^ (min)						
	Control period	3,183	718	11.81	**-**	
	Pandemic	1,260	1,223	40.29	**-**	<0.001
**Access block total time aggregate**^c^ **(hours)**						
	**Control period**	**3,183**	**-**	**-**	**5,420**^c^	
	**Pandemic**	**1,260**	**-**	**-**	**22,653**^c^	**-**
**Boarding time per patient**^b^ **(min)**						
	**Control period**	**3,183**	**640**	**13.42**	**-**	
	**Pandemic**	**1,260**	**1150**	**45.35**	**-**	**<0.001**
**Boarding total time aggregate**^c^ **(hours)**						
	**Control period**	**3,183**	**-**	**-**	**6,970**^c^	
	**Pandemic**	**1,260**	**-**	**-**	**25,954**^c^	

* The considered pandemic period was February 21–May 1, 2020. The control period was the sum of the timespans January 1–May 1, 2018; January 1–May 1, 2019; and January 1–February 20, 2020.

a t-test.

b Mean calculated only for hospitalized patients.

c Access block total time and boarding total time calculated only for hospitalized patients; by definition, it is not an average but the sum of each patient’s access block times. Access block total time and boarding total time ware calculated from February 21–May 1, 2020 for the pandemic period and as the mean of the periods February 21–May 1, 2019, and February 21–May 1, 2018 for the control period.

*Min*, minute; *LOS*, length of stay.

**Table 5:** Risk of overtime for selected time variables accounting for crowding, by period.

	Period*	OR^a^	95% Confidence interval	P
LOS				
	Control period	1.00 (Ref.)	-	
	Pandemic	2.58	2.40–2.78	<0.001
Boarding				
	Control period	1.00 (Ref.)	-	
	Pandemic	2.67	2.46–2.89	<0.001
Access block				
	Control period	1.00 (Ref.)	-	
	Pandemic	2.52	2.33–2.72	<0.001

* The considered pandemic period was February 21–May 1, 2020. The control period was the sum of the timespans January 1–May 1, 2018; January 1–May 1, 2019; and January 1–February 20, 2020.

a ORs estimated by multiple regression analysis adjusted by age, gender, priority code at triage, presence of fever or respiratory symptoms. and need for moderate to high-intensity care.

*LOS*, length of stay; *OR*, odds ratio.
