# Supplementary material for: Impact of Coronavirus Disease 2019 Pandemic on Crowding: A Call to Action for Effective Solutions to “Access Block”
Source: West J Emerg Med. 2021 Jul 16;22(4):860–70. doi: 10.5811/westjem.2021.2.49611 (PMC8328174; doi:10.5811/westjem.2021.2.49611)
Supplement: Supplementary file 1 [file wjem-22-860-s001.docx]

**Table S1.** Wait time (min) by priority code at triage and period.

| **Code** | **Period*** | **Observations** | **Mean** | **Standard error** | **p^a^** |
| --- | --- | --- | --- | --- | --- |
| 5 | Control period | 3,623 | 75 | 1.28 |  |
|  | Pandemic | 293 | 63 | 4.65 | 0.015 |
|  |  |  |  |  |  |
| 4 | Control period | 31,702 | 95 | 0.49 |  |
|  | Pandemic | 3,946 | 78 | 1.43 | <0.001 |
|  |  |  |  |  |  |
| 3 | Control period | 3,112 | 42 | 0.86 |  |
|  | Pandemic | 392 | 39 | 2.57 | 0.342 |
|  |  |  |  |  |  |
| 2 | Control period | 12,130 | 69 | 0.63 |  |
|  | Pandemic | 1,932 | 51 | 1.36 | <0.001 |
|  |  |  |  |  |  |
| 1 | Control period | 807 | 7 | 0.39 |  |
|  | Pandemic | 162 | 10 | 1.38 | <0.001 |

*The considered pandemic period was February 21 to May 1, 2020. The control period was the sum of the timespans January 1 to May 1, 2018; January 1 to May 1, 2019; and January 1 to February 20, 2020; ^a^t test.
